# A role for Regulator of G protein Signaling-12 (RGS12) in the balance between myoblast proliferation and differentiation

**DOI:** 10.1371/journal.pone.0216167

**Published:** 2019-08-13

**Authors:** Adam B. Schroer, Junaith S. Mohamed, Melinda D. Willard, Vincent Setola, Emily Oestreich, David P. Siderovski

**Affiliations:** 1 Department of Physiology & Pharmacology, WVU School of Medicine, West Virginia University, Morgantown, WV, United States of America; 2 Division of Exercise Physiology, West Virginia University, Morgantown, WV, United States of America; 3 Department of Pharmacology, The University of North Carolina, Chapel Hill, NC, United States of America; 4 Department of Neuroscience, West Virginia University, Morgantown, WV, United States of America; University of Rome La Sapienza, ITALY

## Abstract

Regulators of G Protein Signaling (RGS proteins) inhibit G protein-coupled receptor (GPCR) signaling by accelerating the GTP hydrolysis rate of activated Gα subunits. Some RGS proteins exert additional signal modulatory functions, and RGS12 is one such protein, with five additional, functional domains: a PDZ domain, a phosphotyrosine-binding domain, two Ras-binding domains, and a Gα·GDP-binding GoLoco motif. RGS12 expression is temporospatially regulated in developing mouse embryos, with notable expression in somites and developing skeletal muscle. We therefore examined whether RGS12 is involved in the skeletal muscle myogenic program. In the adult mouse, RGS12 is expressed in the tibialis anterior (TA) muscle, and its expression is increased early after cardiotoxin-induced injury, suggesting a role in muscle regeneration. Consistent with a potential role in coordinating myogenic signals, RGS12 is also expressed in primary myoblasts; as these cells undergo differentiation and fusion into myotubes, RGS12 protein abundance is reduced. Myoblasts isolated from mice lacking *Rgs12* expression have an impaired ability to differentiate into myotubes *ex vivo*, suggesting that RGS12 may play a role as a modulator/switch for differentiation. We also assessed the muscle regenerative capacity of mice conditionally deficient in skeletal muscle *Rgs12* expression (via *Pax7*-driven Cre recombinase expression), following cardiotoxin-induced damage to the TA muscle. Eight days post-damage, mice lacking RGS12 in skeletal muscle had attenuated repair of muscle fibers. However, when mice lacking skeletal muscle expression of *Rgs12* were cross-bred with *mdx* mice (a model of human Duchenne muscular dystrophy), no increase in muscle degeneration was observed over time. These data support the hypothesis that RGS12 plays a role in coordinating signals during the myogenic program in select circumstances, but loss of the protein may be compensated for within model syndromes of prolonged bouts of muscle damage and repair.

## Introduction

Regulators of G protein Signaling (RGS proteins) are intracellular GTPase-accelerating proteins (GAPs) that attenuate the G protein-dependent signals that cells receive from their external environment [1, 2]. The RGS protein family member RGS12 is unique in interacting with multiple signaling pathways, including those associated with growth and survival cues from receptor tyrosine kinases (RTKs) and mitogen-activated protein kinases (MAPKs), G protein-coupled receptors (GPCRs), and Ras GTPases [3–9]. It was previously reported that skeletal muscles of developing mouse embryos express RGS12 [10], suggesting a potential role for this unique RGS family member in the skeletal muscle developmental process; however, little has since been reported regarding potential function(s) of RGS12 in the signaling pathways underlying the myogenesis program active during both development and muscle repair. With regards to the latter, adult skeletal muscle has a remarkable regenerative capacity, largely made possible by a specialized population of stem cells—satellite cells—found in a characteristic niche between the sarcolemma and basal lamina of myofibers [11–13]. Myogenesis requires strictly regulated signaling events involving the activation (and subsequent proliferation) of quiescent satellite cells, expression of muscle-specific genes, and differentiation into new muscle fibers during repair or fusion into existing fibers during growth [14]. Muscle regeneration begins with satellite cell activation by alterations to their niche and by factors released during injury, resulting in MYF5 and MYOD induction and several cycles of proliferation. Although some activated satellite cells remain in their niche and return to quiescence as a reservoir, other daughter cells migrate along the sarcolemma then differentiate and fuse with either damaged fibers or with other myoblasts to form repaired or *de novo* myofibers, respectively. This process is characterized by PAX7 down-regulation and up-regulation of muscle-specific genes (*e*.*g*., MRF4, myogenin) [15].

Myogenesis during both embryonic development and adult muscle regeneration shares many regulatory mechanisms [12], including signals from sequential MAPK activation [16]. PAX7-positive satellite cells are constitutively active during embryonic development, but these cells exit the cell cycle during adulthood and become quiescent [17]. External stimuli, such as serum and certain growth factors, can activate satellite cells and promote proliferation by signaling through various MAPK pathways, including extracellular-signal related kinase (ERK1/2) activity [18]. In cultured myoblasts, the onset of differentiation by serum deprivation triggers a decline in ERK1/2 activity [19, 20]. Myoblast differentiation is inhibited by serum and certain growth factors known to activate the small GTPase H-Ras [21, 22]. Various mutations in human rhabdomyosarcoma cell lines (*e*.*g*., RD cells) typically cause an inability to differentiate in response to serum deprivation; multiple mutations in RD cells, including mutation of the *NRAS* gene [23], impair differentiation. Similarly, expression of oncogenic (constitutively-active) H-Ras in myoblasts prevents myotube formation and blocks induction of myogenic genes and muscle-regulatory factors, such as *MyoD1* and myogenin [24–26].

As the ERK1/2 MAPK cascade is activated by many stimuli, multiple mechanisms exist to ensure specific and appropriate biological outcomes, particularly in such a highly temporally regulated process as myogenesis [27, 28]. In this regard, scaffold proteins play an important role by spatially focusing MAPK signaling in many cell systems [29, 30]. RGS12 shares features with such MAPK scaffolds, containing two Ras-binding domains and a GoLoco motif, the latter being a second Gα_i_ binding site that inhibits nucleotide release [31] and facilitates endosomal targeting [6]. RGS12 also has PDZ and PTB domains, each of which promotes additional protein-protein interactions. As previously reported [7], RGS12 uses these latter two domains to interact with multiple components of the Ras/Raf/MEK/ERK1/2 signaling cascade. Here, we employed genetic manipulations of both the C2C12 myoblastic cell line and the mouse genome to investigate the role of RGS12 and its MAPK scaffolding function in the signaling that balances myoblast proliferation *versus* differentiation, both *in vitro* and *in vivo*. Our present studies utilized two, recently described strains of RGS12-deficient mice: namely, constitutive *Rgs12*^*Δ5-8/Δ5–8*^ and Cre recombinase-dependent *Rgs12*^*Δ5-6/Δ5–6*^ knockout strains [32].

## Materials and methods

### Materials

pLKO.1 plasmids encoding mouse *Rgs12*-directed shRNA (IDs TRCN0000037205 [“#2”] and TRCN0000037208 [“#5”]; generated by The RNAi Consortium and purchased from Open Biosystems) were prepared from bacterial stocks via plasmid DNA maxiprep (Qiagen; Valencia, CA) and packaged as lentiviridae by the UNC Lineberger Lenti-shRNA Core Facility (Dr. Tal Kafri, director). A control empty lentiviral vector (Open Biosystems catalog #RHS4080) was also packaged to establish negative control cell lines. GFP-tagged Cre recombinase- or GFP-transducing (negative control) adeno-associated viruses (AAV1.CMV.HI.GFP-Cre.WPRE.SV40 and AAV1.CMV.PI.EGFP.WPRE.bGH, respectively) were purchased from the U. Penn. Virology Core Facility (Philadelphia, PA). Myc-H-Ras(G12V) expression vector was a kind gift from Dr. Channing Der (UNC-Chapel Hill). Cardiotoxin (CTX) from *Naja mossambica mossambica* (Sigma-Aldrich) was dissolved in sterile saline to a final concentration of 10 μM and aliquoted and stored at –20°C. Anti-RGS12 (UNC60-80.4.1 and UNC60-26.2.1; each used at 1:1000 dilution), anti-Pax7 (PAX7; 1:100), anti-myogenin (PCRP-MYOG-1C5; 1:1000), and anti-MHC (MF 20; 1:1000) antibodies were each obtained from the Developmental Studies Hybridoma Bank (Iowa City, Iowa); anti-APPL1 (H-96/sc-67402; 1:500), anti-GAPDH (sc-32233; 1:2000), and anti-Rab9 (G-5/sc-74482; 1:800) antibodies were procured from Santa Cruz Biotechnology Inc (Santa Cruz, CA). Antibodies directed toward cyclophilin A (#2175; 1:1000), total ERK1/2 (p44/42 MAPK; #9102; 1:1000), and phosphorylated ERK1/2 (Thr202/Tyr204; #9101; 1:1000) were obtained from Cell Signaling Technology. Anti-FLAG-epitope (F3165; 1:2000), anti-β-tubulin (T8328; 1:5000), and anti-β-actin (A5441; 1:4000) antibodies were purchased from Sigma-Aldrich; anti-HA-epitope antibody (12CA5; 1:2000) and anti-Myc-epitope antibody (9E10; 1:4000) were obtained from Roche. Anti-H-Ras antibody 146-3E4 from Quality Biotech (Camden, NJ) was a kind gift from Dr. Adrienne Cox (UNC-Chapel Hill) and used at 1:1000 in immunoblotting. Anti-Myf5 antibody (MABE485; 1:1000) was obtained from Millipore. Wheat germ agglutinin (WGA) conjugated to Alexa fluor-488 (Invitrogen) was dissolved in PBS, aliquoted, and stored at -20°C. Two cell lines were obtained from the American Type Culture Collection (ATCC; Manassas, VA): the C2C12 adherent myoblastic cell line (ATCC CRL-1772) derived from a C3H mouse; and, the RD adherent cell line (ATCC CCL-136) derived from a rhabdomyosarcoma of a 7-year-old female Caucasian and exhibiting an unstable karyotype (*i*.*e*., hyperdiploid with a bimodal stemline number of 49 and 50 chromosomes). Precast Tris-glycine SDS-PAGE gels and ancillary reagents/buffers for immunoblotting were obtained from Novex/Invitrogen. Unless elsewhere specified, all additional reagents were of the highest quality obtainable from Fisher Scientific (Pittsburgh, PA).

### Animals

Generation of two independent strains of *Rgs12*-deficient mice (constitutive *Rgs12*^*Δ5-8/Δ5–8*^ and *Rgs12*^*fl/fl*^) was recently described in Gross *et al*. [32]. To generate mice conditionally lacking RGS12 in satellite cells, *Rgs12*^fl/fl^ mice were bred with *Pax7*::Cre mice, purchased from The Jackson Laboratory (*Pax7*^tm1(cre)Mrc^/J; Stock No: 010530). Both mouse strains were maintained in the C57BL/6J background. C57BL/10ScSn-*Dmd*^mdx^ mice were also purchased from The Jackson Laboratory (Stock No: 001801). Female *Dmd*^mdx/mdx^ mice were bred with male homozygous *Rgs12*^fl/fl^; *Pax7*::Cre mice to generate hetero-/hemizygous male *Rgs12*^+/fl^; *Pax7*::Cre; *Dmd*^mdx/Y^ mice. Male *Rgs12*^+/fl^; *Pax7*::Cre; *Dmd*^mdx/Y^ mice were crossed with female *Dmd*^mdx/mdx^ mice to generate male and female mice hemizygous and homozygous for *Dmd*^mdx^, respectively (hereto referred to collectively as *mdx* mice). Male *Rgs12*^+/fl^; *Pax7*::Cre; *Dmd*^mdx/Y^ and female *Rgs12*^+/fl^; *Pax7*::Cre; *Dmd*^mdx/mdx^ mice were subsequently intercrossed to produce both *mdx* mice conditionally lacking RGS12 in *Pax7*+ satellite cells (*Rgs12*^fl/fl^; *Pax7*::Cre; *Dmd*^mdx^) and control littermate *mdx* mice with the wild-type *Rgs12* allele (*Rgs12*^+/+^; *Pax7*::Cre; *Dmd*^mdx^). Mice were housed under standard temperature, humidity, and lighting conditions (12 h light/12 h dark); all mice were provided food and water *ad libitum*. All experiments were conducted on approved protocols in accordance with the West Virginia University Animal Care and Use Committee and followed the National Institute of Health and AAALAC guidelines for use of rodents in research.

### CTX-induced muscle injury

Intramuscular injection of CTX was performed as previously described [33, 34]. Briefly, mice were anesthetized via inhalation of 2% isoflurane. Mouse legs were shaved and cleaned with alcohol. Hind limb muscle(s) were injected with 100 μl of CTX or sterile saline with a 28-gauge needle. After injection, mice were returned to their home cage and monitored until they fully regained consciousness. Hind limb muscle(s) were then harvested at the indicated time points and processed as described.

### Mouse skeletal muscle harvest, myoblast isolation, and myoblast culture

Skin and fascia were removed from euthanized mice and hind limb muscle(s) isolated (removing excess visible fat tissue). Muscles were either flash frozen for subsequent histological analyses or RNA and protein extraction, or fresh muscles were placed into sterile tissue-culture plasticware under phosphate-buffered saline (PBS) to pool sufficient tissue for myoblast isolation. Mouse primary skeletal muscle tissue was harvested from young mice (aged 2–3 weeks), cultured, and processed for fluorescence-activated cell sorting (FACS; ref. [35]) using previously described techniques [10, 36, 37] as modified below. Briefly, isolated muscle tissue was removed, minced with sterile scissors under sterile conditions, and transferred to new tissue-culture plasticware (6 cm dish) for treatment with collagenase/dispase solution (10 mg/mL Collagenase B [Roche] and 2.5 mM CaCl_2_ in Dispase II solution [Roche]). Tissue was then minced further with sterile #10 scalpel blades; tissue slurry was then processed by two rounds of incubation at 37 ^o^C for twelve minutes with intervening trituration to homogenize mixture, prior to filtration using PBS through a pre-wetted 40-μm nylon-mesh cell strainer (Fisher Scientific). Filtered cells were pelleted by centrifugation for 5 min at 1,000 x g, resuspended in Ham’s Complete medium (Ham’s F-10 medium [Gibco] containing 20% fetal bovine serum [FBS], 2.5 ng/mL basic fibroblast growth factor [bFGF; Sigma], penicillin/streptomycin, and fungizone), triturated through a 20G-gauge needle six times, and plated for 2 hours at 37 ^o^C and 5% CO_2_ to remove fibroblast contamination, before being re-pelleted by centrifugation and transferred to collagen-coated tissue-culture plasticware (rat tail collagen obtained from Roche) in fresh Ham’s Complete medium. Cultures were re-fed Ham’s Complete medium every 2 days and passaged before reaching 80% confluency. Cultures to be tested for *ex vivo* differentiation were plated at a higher density (80%) and transferred into Dulbecco’s modified Earle’s medium (DMEM; Gibco) supplemented with 2% horse serum and penicillin/streptomycin. Cultures of primary myoblasts from conditional *Rgs12*-knockout mice were maintained in Ham’s Complete medium and infected overnight with Cre- (or GFP-only) expressing AAV prior to harvest and lysis for immunoblotting.

### Immunoblotting

Cells and tissue were lysed in ice-cold RIPA buffer with protease inhibitors (Roche). Tissues were minced in a 1.5 mL Eppendorf tube prior to homogenization using a bench top rotor stator. Cell and tissue lysates were sonicated on ice prior to being clarified by centrifugation at 20,000 X g for 15 minutes. Protein content was quantified by the Bradford protein assay using BSA as a standard. Lysates were diluted in Laemmli buffer (BioRad, Hercules, CA) and resolved on 4–16% pre-cast SDS-polyacyrlamide gels (BioRad), transferred to nitrocellulose membranes, immunoblotted with primary (overnight, 4°C) and HRP-conjugated secondary antibodies (*e*.*g*., Sigma #A5278 at 1:1000 dilution, 1 h at room temperature), and visualized by chemiluminescence (Pierce ECL Substrate or SuperSignal West Dura Extended Duration Substrate).

### RNA extraction and quantitative real-time polymerase chain reaction (qRT-PCR) procedure

RNA extraction and qRT-PCR procedures were previously described by Schroer *et al*. [38]. Briefly, TRIzol reagent (Invitrogen) was used to extract RNA from both tissue and cells. Muscle tissue was minced and homogenized using a bench top rotor stator. RNA was extracted according to manufacturer’s instructions using the Direct-zol RNA MiniPrep kit from Zymo Research. Reverse transcription and genomic DNA elimination was performed using the QuantiTect Reverse Transcription Kit from Qiagen. Two microliters of cDNA were used in each PCR reaction using the QuantiTect SYBR Green PCR Master Mix (Qiagen). Primers (10 μM) were designed for *Rgs12* mRNA (spanning exons 5–6) sequence elements (fwd primer: 5′-CATCAGCAACAACAGCCTGA-3′; rev primer 5′-CATGACTGAAGCACTCACAGG-3′). Primer sequences for eMHC were described previously [39] (fwd primer: 5’-AAAAGGCCATCACTGACGC-3’; rev primer: 5’-CAGCTCTCTGATCCGTGTCTC-3’) and were purchased from ThermoFisher Scientific. RT^2^ qPCR Primer Assay kits for mouse 18S rRNA and *Gapdh* were purchased from Qiagen. PCR was performed in a Qiagen Rotor Gene-Q system (initial 15 min denaturation at 95°C followed by 40 cycles of 15 s denaturation at 95°C, 30 s annealing at 60°C, and 30 s extension at 72°C). We utilized the SYBR green dye qPCR technique to detect double-stranded PCR amplicons as they accumulated during PCR cycling. Melting curves were obtained after each qRT-PCR experiment to assure specificity of resultant amplicons. Relative quantification of gene expression was performed using the ΔΔC_t_ method [40] with 18s rRNA and *Gapdh* chosen as stable housekeeping genes.

### Immunofluorescence analyses

Cultures of adherent cells were grown on glass coverslips, fixed in PBS containing 4% paraformaldehyde, permeabilized in PBS containing 0.1% Triton X-100, and then stained using primary and fluor-conjugated secondary antibodies (and often with DAPI as a nuclear DNA stain) as indicated in the Results section and Figure legends. Stained cell cultures were then bonded in Vectashield and imaged using epifluorescence on an IX70 Olympus microscope. Single-color images were pseudocolored and overlaid in Photoshop (CS6, Adobe).

### Lipid dot-blot protein overlay assay

Recombinant *Schistosoma japonica* glutathione-S-transferase (GST), and GST fusions with the N-terminal PDZ and PTB domains of RGS12 (*i*.*e*., amino-acids 9 to 406 of UniProt O08774), were separately expressed in *E*. *coli* and purified by high-pressure liquid chromatography (HPLC) as per previously published protocols [41]. Each purified protein was incubated (at a final concentration of 20 μg/mL protein within blocking buffer of 0.1% Tween-20 and 3% BSA in phosphate-buffered saline) for 1 hour at room temperature with a lipid dot-blot membrane (PIP Strip; cat #P-6001 from Echelon Biosciences, Salt Lake City UT) that had been pre-blocked by overnight incubation in blocking buffer at 4 ^o^C with gentle agitation as per manufacturer’s protocol. After three consecutive, ten-minute washes with blocking buffer lacking BSA, membranes were incubated with anti-GST monoclonal antibody (Sigma #G1160; 1:2000 dilution in blocking buffer), washed again three times, incubated with anti-mouse horseradish peroxidase (HRP) secondary antibody (Sigma #A5278; 1:2000 dilution in blocking buffer), washed again three times, and then protein/lipid-spot interactions detected using the same chemiluminescence protocol as used for immunoblotting described above.

### Stable over-expression of oncogenic H-Ras in the myoblastic C2C12 cell line

Cultures of the parental C2C12 myoblastic cell line were transfected with expression vector DNA (encoding myc-tagged H-Ras(G12V); a gift from UNC colleague Dr. Channing Der) and Lipofectamine 2000 (Invitrogen) as per manufacturer’s instructions. Transfected cells were then selected in culture medium containing 10% FBS and 1 mg/mL hygromycin for two weeks, prior to clonal dilution, expansion under constant drug selection, and screening of whole cell lysates via immunoblotting with anti-Myc epitope antibody 9E10.

### Co-immunoprecipitation

Protein co-immunoprecipitation experiments were conducted using previously described techniques [37] and reagents [6, 7].

### Stable RGS12 over-expression in the myoblastic C2C12 cell line

Cultures of the C2C12 cell line were separately transfected with a control yellow-fluorescent protein (YFP) expression vector or either an HA epitope-tagged or Flag epitope-tagged, full-length RGS12 expression construct [6, 7], using Lipofectamine 2000 (Invitrogen) as per manufacturer’s directions. Twenty-four hours later, cells were cultured in drug-selection medium (GM + 1 mg/mL G418 for Flag-RGS12; GM + 1 mg/mL hygromycin for HA-RGS12) for two weeks. Stably-selected C2C12 cell populations were switched from GM (DMEM + 10% FBS) to DM (DMEM + 2% HS) for two days. To quantify differentiation, cell cultures were then fixed with para-formaldehyde, permeabilized, and stained with a primary antibody to sarcomere myosin heavy chain MHC (DSHB cat# MF 20; Antibody Registry ID AB_2147781) and Alexa-fluor 594 secondary antibody (Molecular Probes); nuclei were counterstained with DAPI. Myotube formation within derived C2C12 cell line cultures was quantitated by calculation of the fusion index, as previously described [42]. Briefly, nuclei from MHC-positive, multi-nucleated cells and MHC-negative, non-fused cells were separately counted using ImageJ software and the fusion index calculated as the ratio of nuclei present in fused MHC-positive cells to the total number of nuclei in the field (expressed as a percentage). Mock transfected and myc-RGS12-overexpressing cultures of C2C12 cells were incubated with EdU for 60 minutes, to obtain proliferation indices, and click-iT labeling and DNA counterstaining was performed directly as per manufacturer’s protocol (Click-iT EdU Alexa Fluor 488 kit; ThermoFisher). The percentage of Alexa fluor 488-positive cells was assessed using ImageJ software.

### Stable knockdown of RGS12 expression in the myoblastic C2C12 cell line

To create C2C12 cells with stable decreases in RGS12 protein expression, cultures of the parental C2C12 line were infected with lentiviridae encoding either a non-specific control shRNA or one of two different shRNAs targeting *Rgs12* (#2, #5) and then selected in puromycin-containing growth medium (DMEM + 10% FBS) for two weeks. Cell cultures were then switched from growth medium to differentiation medium (DMEM + 2% HS) for five days; as a positive control for low-to-nil fusion index, the poorly-differentiating, human RD cell line was also cultured for five days in differentiation medium (rightmost panel). Quantification of C2C12 cell line differentiation, via the fusion index, was performed as described above.

### RGS12 over-expression and myotube formation assays in primary mouse myoblasts

Cultures of primary mouse myoblasts were separately transfected with a control (empty pcDNA3.1 vector) or HA epitope-tagged full-length RGS12 expression construct [6, 7], using Lipofectamine 2000 (Invitrogen) as per manufacturer’s directions. Cultures of primary mouse myoblasts at 80% confluence were switched from GM (Ham’s F-10 + 20% FBS) to DM (DMEM + 2% HS) for 48 hours. Post-differentiation, cell cultures were fixed with para-formaldehyde, permeabilized, and stained with a primary antibody to sarcomere myosin heavy chain MHC (DSHB cat# MF 20; Antibody Registry ID AB_2147781) and Alexa-fluor 594 secondary antibody (Molecular Probes); nuclei were counterstained with DAPI. Myotube formation in primary myoblast cultures was quantitated using fusion index: nuclei from MHC-positive, multi-nucleated cells and MHC-negative, non-fused cells were separately counted using ImageJ software and the fusion index calculated as the ratio of nuclei present in fused MHC-positive cells to the total number of nuclei in the field (expressed as a percentage) Three separate fields were counted for each condition.

### Histological analysis of skeletal muscle

Skeletal muscles were excised from mice and placed in optimum cutting temperature (OCT) embedding compound (Sakura Finetech) prior to being flash frozen. Eight μm thick transverse sections of muscle were sliced using a cryostat and placed on Superfrost Microscope Slides (Fisherbrand). Tissues sections were stained with hematoxylin and eosin (H&E) and examined under a bright-field microscope (EVOS FL Auto). To assess skeletal muscle cross-sectional area (CSA), sections were labeled with WGA-conjugated Alexa fluor-488 (10 μg/mL). Tissue sections were fixed in 4% paraformaldehyde for 15 min at room temperature. Sections were incubated at room temperature for 60 minutes with WGA-conjugated Alexa fluor-488. Sections were subsequently permeabilized with 0.3% Triton X-100 and slides were cover-slipped with Vectashield Hardset Mounting Media containing DAPI. Sections were examined with a fluorescent microscope (EVOS FL Auto) and scanning images of the sections were recorded. The CSA of each myofiber was measured using the free-hand tracing tool within the EVOS FL Auto software. At least 800 myofibers were analyzed per mouse.

### *In vivo* isometric force measurements

Plantar flexor muscle function was assessed as described previously [43]. Briefly, mice were anesthetized by inhalation of a mixture of 97% oxygen and 3% isoflurane gas and placed supine on a plate heated to 37°C to maintain body temperature. The hindlimb was then immobilized with the ankle positioned at 90° flexion and secured to the footplate of the dynamometer (model 6350*358; Cambridge Technology, Aurora Scientific, Aurora, ON, Canada). Subcutaneous platinum electrodes were placed on either side of the tibial nerve to activate plantar flexor muscles. Maximal force was measured by seven sequential electrical impulses (single pulse, 10, 20, 50, 75, 100, and 120 Hz) with five min of rest between each contraction. Contractile data were analyzed off-line (Dynamic Muscle Analysis software; Aurora Scientific). The maximum tension generated by the CTX injured leg was normalized to the sham PBS injected leg of the same animal and recorded as percent recovery (*i*.*e*., (CTX tension / PBS tension) *100%). The tension generated by *mdx* mice was normalized to body mass to account for differences in mouse strains.

## Results and discussion

### Expression of *Rgs12* in mouse skeletal muscle, satellite cells, injured muscle, and muscle-derived cell lines

**Fig 1:**
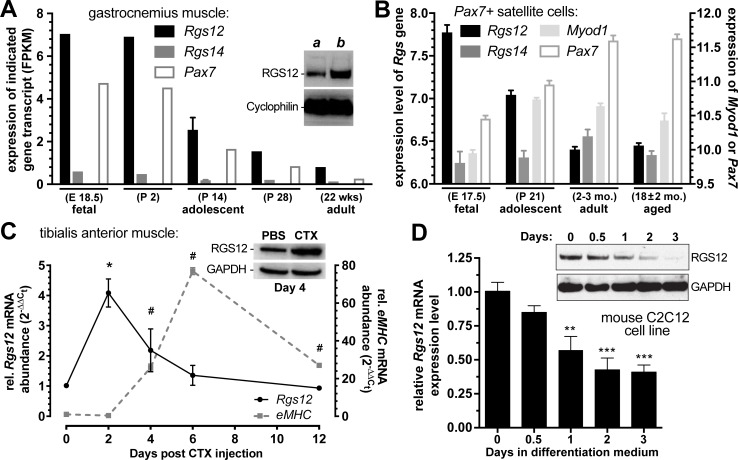
*Rgs12* expression is high in mouse embryonic skeletal muscle and active satellite cells, upregulated early during muscle repair, and downregulated upon differentiation of the myoblastic C2C12 cell line. **(A**) Relative expression (mean ± SEM of Fragments Per Kilobase of transcript per Million mapped reads [FPKM]) of *Rgs12*, *Rgs14*, and *Pax7* gene transcripts within indicated ages of mouse gastrocnemius muscle samples was obtained via RNA-Seq using an Illumina HiSeq 2000. Based on Gene Expression Omnibus dataset GSE108402 [50]. *Inset*, detection of RGS12 protein expression via immunoblotting of **(*a*)** 100 μg of whole gastrocnemius muscle lysate from a 3-month-old C57BL/6J mouse and **(*b*)** 50 μg of lysate from myoblasts isolated from cardiotoxin-injected tibialis anterior (TA) muscle. **(B)** Normalized expression levels (mean ± SEM) of *Rgs12*, *Rgs14*, and *Pax7* gene transcripts within indicated ages of flow-sorted, *Pax7*+ mouse skeletal muscle satellite cells were obtained via an Affymetrix Mouse Gene 1.0 ST microarray, based on Gene Expression Omnibus dataset GSE47401; published in [51]. **(C)** Relative expression of *Rgs12* and *eMHC* in TA muscle following CTX-induced muscle damage. RNA was extracted from muscle at the indicated time points and quantified using qRT-PCR, with *Gapdh* abundance as an internal control. *, p < 0.01 *Rgs12* level compared to time zero (one-way ANOVA with Dunnett’s test); #, p < 0.0001 *eMHC* level compared to time zero (one-way ANOVA with Dunnett’s test). *Inset*, TA muscle was injected with 0.1 ml of 10 μM cardiotoxin (CTX) diluted in PBS; contralateral, PBS-injected TA muscle was used as a control. After four days, the muscles were harvested and used for RGS12 protein expression analysis by immunoblotting (with GAPDH protein levels interrogated in parallel as a loading control). **(D)** C2C12 cell line cultures (4 x 10^5^ cells/well) were maintained in growth medium (DMEM containing 10% fetal bovine serum [FBS]) for two days. Differentiation was induced by replacing growth medium with differentiation medium (DMEM containing 2% horse serum [HS] instead of FBS). Total RNA and protein lysates were separately isolated from cell cultures at the indicated time points (hours) after the switch to differentiation medium. *Rgs12* mRNA and RGS12 protein levels were determined by qRT-PCR and immunoblotting, respectively. GAPDH mRNA and protein levels were used as internal controls for each experiment. **, p < 0.01; ***, p < 0.001 *versus* level observed at time zero (one-way ANOVA with Dunnett’s test).

Confirming our prior findings of skeletal muscle expression of RGS12 beginning at embryonic day 9.5 during mouse development [10], more recent data [44] from whole transcriptome shotgun sequencing (RNA-Seq) of mouse gastrocnemius muscle-derived RNA from various developmental stages indicate that *Rgs12* is expressed in developing skeletal muscle during fetal and early postnatal time points, and this expression fades over time in mature adult skeletal muscle (Fig 1A; Gene Expression Omnibus GSE108402). In microarray-based expression studies, *Rgs12* is also observed to be expressed in *Pax7*-positive satellite cells from embryonic day E17.5 mice [45]; while still detectable in adult mice, *Rgs12* expression decreases over time in the *Pax7*-positive satellite cell population (Fig 1B), coinciding with the typical timing of satellite cells moving to a quiescent state [46]. In contrast, *Rgs14*, the closest paralog to *Rgs12* and itself a MAPK scaffold [37, 47], is expressed at a lower level than *Rgs12*; furthermore, *Rgs14* expression levels are relatively unaltered by age (Fig 1A and 1B).

In addition to being elevated during embryonic myogenesis, *Rgs12* expression was also increased during adult myogenesis. Specifically, *Rgs12* transcript is seen transiently upregulated in TA muscle undergoing repair: *i*.*e*., four-fold increased two days following CTX-induced injury, then returning to levels equivalent to uninjured muscle four days after CTX injection (Fig 1C). In contrast, the embryonic form of myosin heavy chain (eMHC), a differentiation marker of regenerating myofibers [34, 48], was not observed to be upregulated until four days after CTX-induced injury, and remained elevated 12 days after CTX injection, indicating that *Rgs12* levels are downregulated upon the differentiation and maturation of regenerating myofibers.

To investigate whether observed decreases in *Rgs12* expression over time are directly associated with the transition from myoblast proliferation to differentiation, we examined *Rgs12* mRNA and RGS12 protein levels in the C2C12 myoblastic cell line, a commonly used *in vitro* model [49] that can be maintained in a proliferative state by culture in 10% fetal bovine serum, or rapidly switched toward differentiation into myotubes upon culturing them instead in 2% horse serum. By 24 hours after the switch to differentiation medium, *Rgs12* transcript levels were observed to drop to 50% compared with proliferating C2C12 cells (Fig 1D); by 72 hours after the switch to differentiation medium, RGS12 protein expression was barely detectable by immunoblotting (Fig 1D). These findings suggest that RGS12 expression declines during myoblast differentiation.

### RGS12 is associated with activated H-Ras and early (but not late) endosomes in C2C12 cells

**Fig 2:**
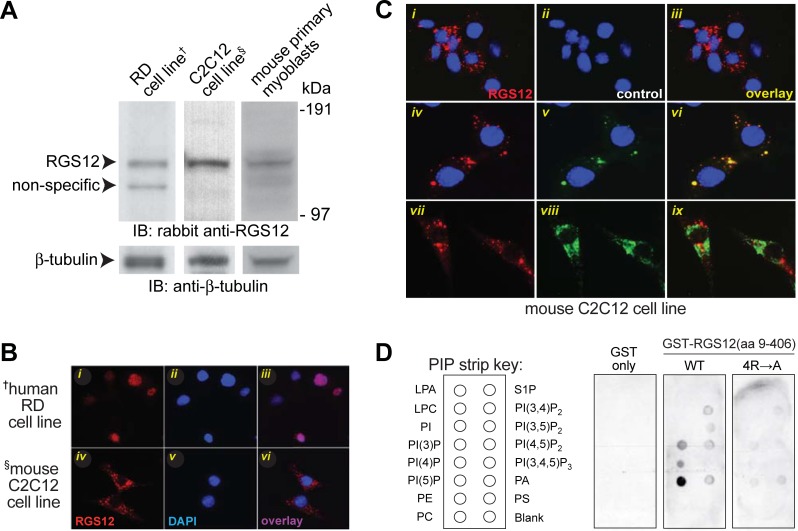
RGS12 expression is observed in cytosolic puncta within the myoblastic C2C12 cell line, but localized in the nucleus in the poorly differentiating human RD cell line. **(A)** Immunoblotting (IB) of cell lysates from all three cell types indicates RGS12 protein expression, as detected using a rabbit anti-RGS12 polyclonal antibody previously described [7]; β-tubulin protein levels were also examined by immunoblotting in parallel as a loading control. **(B)** Cultures of the poorly differentiating, human rhabdomysarcoma RD cell line and the more-easily differentiated, mouse C2C12 cell line were separately fixed and stained with DAPI; RGS12 protein was detected by indirect immunofluorescence using UNC60-26.2.1. Panels (i-vi) represent: (i, iv) anti-RGS12 antibody detection with Alexa-fluor-594 secondary antibody; (ii, v) DAPI nuclear stain; and, (iii, vi) overlay of both images. **(C)** Endogenous RGS12 localizes to early (APPL1-positive) endosomes within C2C12 cells. C2C12 cell line cultures (4 x 10^5^ cells/well) were maintained in growth medium (DMEM containing 10% fetal bovine serum [FBS]) for two days. Cells were then fixed in paraformaldehyde, permeabilized, and stained with primary mouse monoclonal antibody UNC60-26.2.1 and secondary Alexa-fluor-594 anti-mouse antibody, alone or in combination with primary anti-APPL1 or -Rab9 rabbit polyclonal antibodies followed by Alexa-fluor-488 secondary anti-rabbit antibody. Overlays in panels (vii-ix) are absent the DAPI nuclear stain (*blue*) to highlight lack of overlap between RGS12 and Rab9 signals. **(D) RGS12 N-terminus binds to select phosphatidylinositides in a lipid dot-blot protein overlay assay.** A “PIP Strip” nitrocellulose membrane pre-spotted with the indicated phospholipid species was probed with 20 μg/mL of GST alone, recombinant GST-RGS12 protein (amino-acids 9–406 spanning PDZ and PTB domains; “WT”), or GST-RGS12(aa 9–406) protein with alanine substitutions to four arginines in the PTB domain (Arg-255, -260, -262, and -308; “4R→A”) previously predicted by electrostatic contouring [61] to be involved in phospholipid binding. After extensive washing, the binding of protein to phospholipid spots was detected by chemiluminescence using anti-GST mouse monoclonal antibody and anti-mouse-horseradish peroxidase conjugated secondary antibody. Lipid abbreviations: LPA, lysophosphatidic acid; LPC, lysophosphocholine; PI, phosphatidylinositol; PE, phosphatidylethanolamine; PC, phosphatidylcholine; S1P, sphingosine-1-phosphate; PA, phosphatidic acid; PS, phosphatidylserine.

RGS12 protein was seen by immunoblotting to be expressed within a human rhabdomyosarcoma RD cell line, proliferating C2C12 cells, and isolated primary myoblasts from mouse gastrocnemius muscle (Fig 2A). Indirect immunofluorescence labeling of RGS12 revealed localization within cytosolic puncta of proliferating C2C12 cells; by contrast, the poorly differentiating human rhabdomyosarcoma RD cell line [52] was observed to express RGS12 predominantly in the nucleus (Fig 2B). A nuclear localization for RGS12 in RD cells likely reflects its reported function as a transcriptional repressor that affects *MNX1* expression and AKT activity [9, 53]; this function is presumably in contrast to (or at least independent of) its established role as an endosomally targeted, G protein/MAPK signalsome scaffold when localized in cytosolic puncta [6, 7, 32, 54, 55].

Given the punctate nature of the RGS12 expression pattern observed in C2C12 cells, we performed dual-label immunofluorescence staining to assess whether RGS12 was potentially associating with early or late endosomes. As shown in Fig 2C, RGS12 expression was coincident with that of the early endosomal marker APPL1 [56], but not that of the late endosomal marker Rab9 [57], in C2C12 cells. This association profile is consistent with our prior observations of RGS12 association with the early endosomal marker EEA1 within PC12 cells treated with nerve growth factor (NGF) to induce neuritogenesis [7]. In addition, APPL1-containing early endosomes have been reported to serve as platforms for MAPK signalsome assembly [58], reinforcing the notion that RGS12 may serve as a MAPK scaffold in myoblasts similar to its function in developing neurons [7]. Endosomal targeting of RGS12 is also consistent with our *in vitro* finding (Fig 2D) that the N-terminal PTB domain of RGS12 interacts with phosphatidylinositol lipids including PtdIns(3)P and PtdIns(5)P, two lipid species known to have roles in intracellular membrane compartments and endosomal trafficking [59, 60]

**Fig 3:**
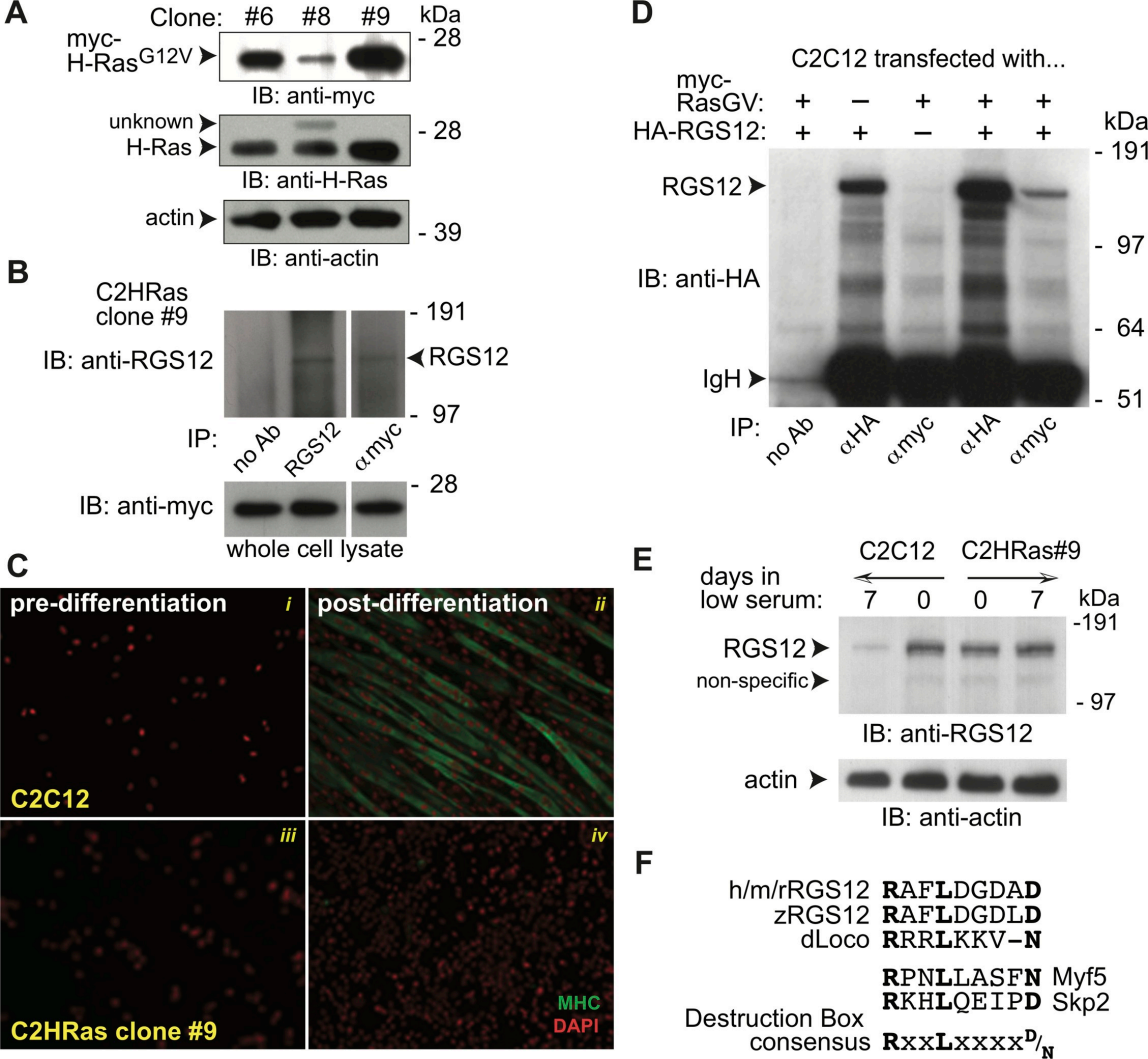
RGS12 interacts with activated H-Ras and is downregulated within the C2C12 cell line during differentiation. **(A)** Immunoblotting (IB) of cell lysates from three stable clones of the C2C12 cell line indicating their stable expression of a constitutively-active, GTPase-deficient (G12V) mutant of H-Ras protein (via detection of its N-terminal myc-epitope tag). **(B)** Co-immunoprecipitation of myc-tagged, activated H-Ras (G12V mutant) with endogenous RGS12 protein expressed in the C2HRas cell line clone 9. IP: immunoprecipitation; IB: immunoblotting. **(C)** To test the effect of stable expression of activated H-Ras on myoblast differentiation *in vitro*, multi-nucleated myotube content of indicated cell populations (either parental C2C12 cells [panels i, ii] or C2HRas clone #9 cells [panels iii, iv]) was measured by fixation and staining for sarcomere myosin (MHC; *green*) and nuclear DNA content (DAPI; pseudocolored *red*), either pre-differentiation (panels i, iii) or post-differentiation by culture for 5 days in low serum (2% horse serum; panels ii, iv). The mean fusion index for several C2C12 cell populations was 40% (± 2.5%; SEM), consistent with other reports [68, 69]. In contrast, the C2HRas cell line clone 9 was incapable of myotube formation (panel iv) and no fusion index could be calculated. **(D)** Co-immunoprecipitation of myc-tagged, activated H-Ras (G12V mutant) with ectopically co-expressed, HA-tagged RGS12 in C2C12 cells. IgH: immunoglobulin heavy-chain. **(E)** Endogenous levels of RGS12 protein are down-regulated during C2C12 differentiation into myotubes (*i*.*e*., 7 days of culture in low serum medium), whereas the same culture conditions did not lower RGS12 levels within the C2HRas cell line clone #9. **(F)** Mammalian RGS12 proteins encode a “destruction box” recognition motif, conforming to the consensus RxxLxxxx(D/N), where “x” is any amino-acid, that is found in most substrates of the Anaphase-Promoting Complex (APC). This destruction box sequence is completely conserved across human (h), mouse (m), and rat (r) RGS12 proteins (i.e., amino acids 402–410 of mouse RGS12: RAFLDGDAD); this consensus motif is preserved in zebrafish (z) and Drosophila (d) RGS12 orthologs, as well as in the APC targets Skp2 and Myf5.

Metastatic rhabdomyosarcoma is a skeletal muscle satellite cell-derived cancer [62] in which oncogenic H-Ras mutations and increased ERK activity have been described [63–65]. As we have previously shown that RGS12 can assemble an activated H-Ras-driven MAPK signalsome within dorsal root ganglion neurons [7], observations of differential RGS12 localizations within cultures of poorly differentiable RD cells *vs* easily differentiable C2C12 cells (Fig 2) prompted us to investigate the relationship between RGS12 and H-Ras in the latter (Fig 3). H-Ras activation (upstream of ERK1/2 activation) promotes proliferation and inhibits myotube formation, an early myogenic step, whereas inactivation of ERK1/2 is necessary for myogenesis to occur [66, 67]. Clones of the C2C12 cell line stably expressing activated H-Ras were therefore established; clone 9, with the highest stable expression of activated H-Ras (*e*.*g*., Fig 3A), was chosen for further characterization, especially given evidence of endogenous RGS12 interaction with the ectopically expressed, oncogenic H-Ras mutant in this stable sub-line (Fig 3B).

### Oncogenic H-Ras expression affects C2C12 differentiation and RGS12 stability

Stable overexpression of oncogenic H-Ras in C2C12 cells has previously been shown to inhibit myotube formation under *in vitro* culture conditions that normally drive myoblast differentiation [24–26]. An established means to assess such myoblast differentiation *in vitro* is measurement of the fusion index [68, 69], which quantifies the presence of sarcomere myosin heavy chain (MHC)-expressing, multi-nucleated myotubes as enumerated by immunofluorescence detection via the MF20 anti-MHC antibody and DAPI nuclear staining, respectively. After 5 days of culture in low serum (differentiation medium), the parental C2C12 line formed MHC-positive myotubes (Fig 3C, panel ii). In contrast, the daughter line expressing oncogenic H-Ras did not form myotubes (Fig 3C, panel iv) and, thus, no fusion index could be calculated.

The oncogenic H-Ras mutant was observed to co-immunoprecipitate with ectopically over-expressed RGS12 in the C2C12 cell line (Fig 3D); in addition, co-expression of RGS12 with oncogenic H-Ras appears to increase the amount of RGS12 expressed upon its transient co-transfection (compare lanes 2 and 4 of Fig 3D). In contrast to the loss of RGS12 levels seen in the parental C2C12 cell line after culturing in differentiation medium (Fig 3E), the C2HRas#9 cell line showed no loss of RGS12 expression when switched to differentiation medium (albeit with constant drug (hygromycin)-selection pressure). A proteasome-dependent path to RGS12 protein degradation during myoblast differentiation may be operative in C2C12 cells, as this would be consistent with findings reported for the *Drosophila* RGS12 ortholog, Loco, which is ubiquitinated and degraded in an Anaphase-Promoting Complex (APC)- and Cdh1-dependent process during glial differentiation in the developing fruit fly [70]. Mammalian RGS12 proteins share a destruction box motif [71] with Skp2 and Myf5 (Fig 3F); Cdh1, a regulatory component of APC, targets Skp2 and Myf5 for degradation, resulting in cell cycle withdrawal and myogenic fusion [72]. Thus, RGS12, like Loco, may be a target of Cdh1 for ubiquitin-mediated degradation during myoblast differentiation.

### Altered expression of RGS12 affects the balance between C2C12 cell proliferation and differentiation

**Fig 4:**
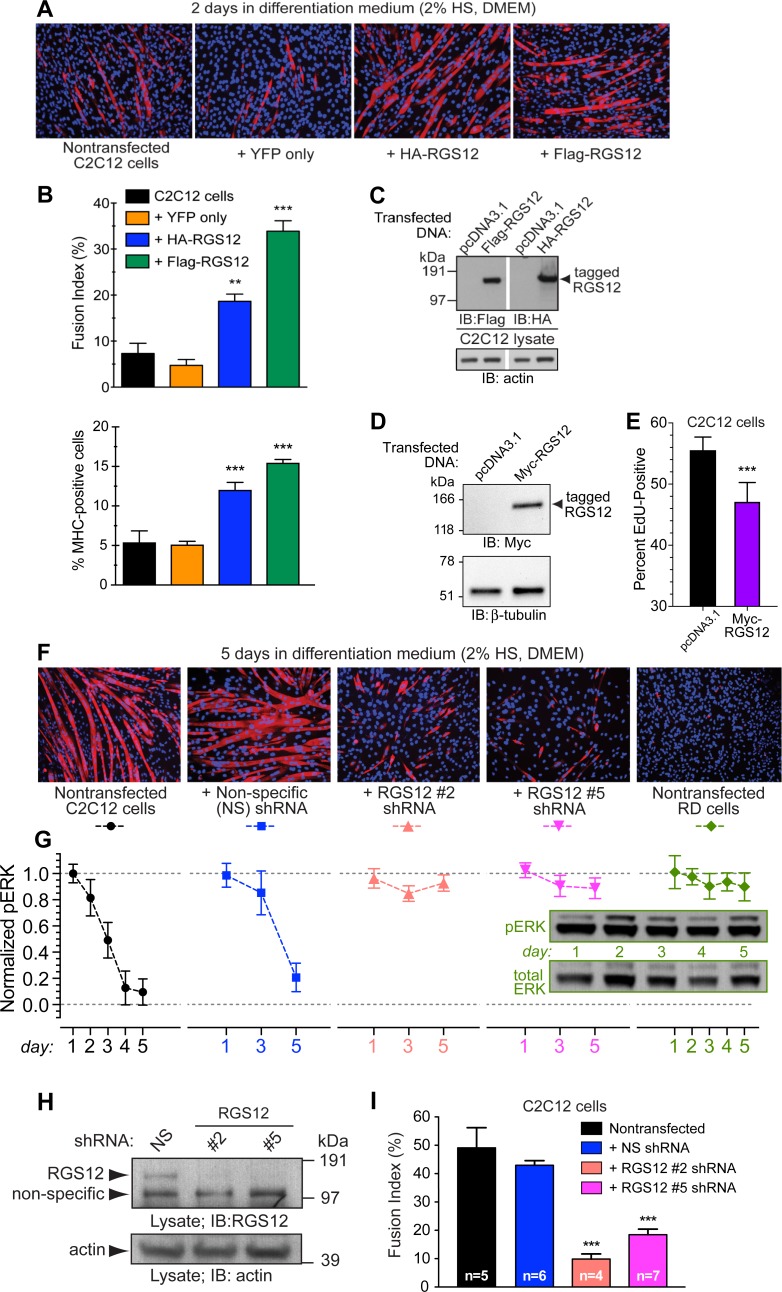
Ectopic RGS12 over- and under-expression affects myotube formation by the C2C12 cell line. **(A)** RGS12-overexpressing and control C2C12 cell line cultures were each switched from growth medium (DMEM, 10% FBS) to differentiation medium (DMEM, 2% horse serum) and cultured for two days. Cells were then fixed with paraformaldehyde and stained with anti-MHC antibody (MF20) and Alexa-fluor-594 secondary antibody (*red*). Nuclei were visualized with DAPI staining (*blue*). **(B)** Differentiation to myotubes was quantitated by the fusion index [36, 42): namely, the percentage of nuclei present in fused, MHC-positive cells *vs* total nuclei in the field. Graphed below the fusion index is the quantitation of MHC-positive cells observed in each field examined. Statistical significance was tested by one-way ANOVA: **, p<0.01; ***, p<0.001. **(C)** Expression of epitope-tagged, full-length RGS12 was confirmed by immunoblotting (IB) with indicated anti-epitope tag antibodies. **(D)** Ectopic expression of Myc-epitope tagged, full-length RGS12 was verified by immunoblotting (IB) of whole cell lysates; equal total protein loading was verified by β-tubulin detection. **(E)** RGS12-overexpressing and mock transfected (empty vector) C2C12 cell line cultures in growth medium (DMEM, 10% FBS) were separately incubated for 60 minutes with 5-ethynyl-2’-deoxyuridine (EdU) to detect DNA synthesis by proliferating cells. Click-iT labeling with Alexa-fluor-488 identified cells with newly synthesized DNA. The percentage of EdU positive cells were counted using ImageJ software. N = 3 independent experiments. Statistical significance was established by Student’s t-test: ***, p<0.001. **(F)** C2C12 cell cultures were infected with lentiviridae encoding either a non-specific (NS) control shRNA or one of two different shRNAs targeting *Rgs12* (#2, #5), and then selected in puromycin-containing growth medium (DMEM + 10% FBS) for two weeks. Cell cultures were then switched from growth medium to differentiation medium (DMEM + 2% HS) for five days; as a positive control for low-to-nil fusion index, the poorly-differentiating, human RD cell line was also cultured for five days in differentiation medium (rightmost panel). Cell cultures were immunolabeled with a primary antibody directed against sarcomere MHC (MF20) and Alexa-fluor-594 secondary antibody; nuclei were counterstained with DAPI. (**G**) Underneath each fluorescence micrograph is the plot of phospho-ERK content (quantified by densitometry and normalized to total ERK content) from parallel cultures harvested at indicated timepoints; immunoblot inset within graph presents representative data from nontransfected RD cells over five days of culture. **(H)** shRNA-mediated knockdown of RGS12 expression was confirmed by immunoblotting of whole cell lysates from indicated, shRNA-expressing C2C12 cell lines; β-actin protein levels were also examined by immunoblotting in parallel as a loading control. **(I)** Myotube formation within indicated C2C12 cell cultures was quantitated by calculation of the fusion index: nuclei from sarcomere MHC-positive, multi-nucleated cells and MHC-negative, non-fused cells were separately counted using ImageJ software and the fusion index calculated as the ratio of nuclei present in fused MHC-positive cells to the total number of nuclei in the field (expressed as a percentage). ***, p < 0.005 versus control shRNA-expressing C2C12 cell line; Student’s t-test.

Given these data suggesting a potential role for regulated RGS12 levels in affecting myoblast cell fate, we examined the effect of ectopic RGS12 over-expression on C2C12 myotube formation. A pronounced increase in myotube formation was observed even after just 2 days of culture in low serum differentiation medium, upon transient overexpression of either HA- or Flag-tagged, full-length RGS12 protein (Fig 4A–4C). Proliferation, as assessed by EdU incorporation, decreased commensurately following RGS12 ectopic over-expression (Fig 4D and 4E).

We next examined the consequences of reducing RGS12 expression in the C2C12 cell line, using lentiviral-mediated transduction of short hairpin RNAs (shRNAs) targeting the *Rgs12* transcript (Fig 4F–4I). Two independent shRNAs, separately targeting either the open-reading frame or 3’ untranslated region of the *Rgs12* transcript, each greatly reduced expression of the ~160 kDa full-length RGS12 protein (Fig 4H) and also greatly reduced the production of myotubes after 5 days of culture in low serum differentiation medium (fusion index quantified in Fig 4I); as expected, culturing the human rhabdomyosarcoma RD cell line for five days in differentiation medium was not observed to induce myotube formation *in vitro* (Fig 4F and 4G, rightmost panels). Phospho-ERK1/2 content was significantly reduced in later days during the five-day treatment with differentiation medium (*e*.*g*., days 4 and 5 for non-transfected C2C12 cells; Fig 4G); this reduction was not evident in RD cells under the same five-day treatment, nor in C2C12 cell lines with effective shRNA-mediated RGS12 knockdown (#2 and #5; Fig 4F–4I).

### Ablation and re-expression of RGS12 both affect the differentiation of explanted mouse primary myoblasts

**Fig 5:**
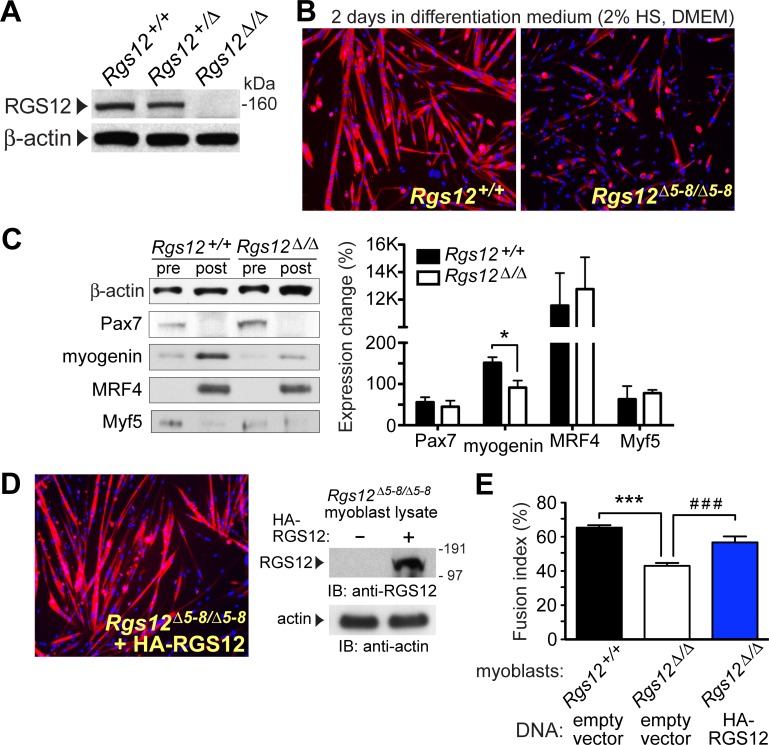
Myotube formation by primary myoblasts isolated from RGS12-null mice is disrupted, but restored upon RGS12 re-expression. **(A)** Confirmation of RGS12 loss in primary myoblasts from *Rgs12*^*Δ5-8/Δ5–8*^ mice via immunoblotting of myoblast lysates with indicated antibodies. Skeletal muscle myoblasts were isolated from tibialis anterior (TA) muscles of 2- to 3-week old homozygous *Rgs12*^*Δ5-8/Δ5–8*^ mice, heterozygous *Rgs12*^*+/Δ5–8*^ mice, or wild-type littermate controls, and cultured in F10 Ham’s medium with 20% FBS and 2.5 ng/mL bFGF prior to lysis. **(B)** Cultures of wild-type myoblasts or *Rgs12*^*Δ5-8/Δ5–8*^ myoblasts were each switched to differentiation medium (DMEM, 2% horse serum) when at 80% confluence and cultured for an additional 2 days. Cell cultures were then fixed, permeabilized, and stained with anti-MHC (MF20; *red*). Nuclei were visualized with DAPI (*blue*). **(C)** Myoblast cultures identical to those of panel B were cultured in differentiation medium for 2 days and lysed (“post” = post-differentiation over 2 days); parallel cultures were treated as in panel A before lysis (“pre” = pre-differentiation). Resultant lysates were immunoblotted with antibodies against indicated proteins (β-actin as a loading control). Below the representative immunoblots is a bar-graph of quantitated protein levels normalized as percentage change in expression between pre- and post-differentiation samples (*, p < 0.05; Student’s t-test). **(D)** Separate *Rgs12*^*Δ5-8/Δ5–8*^ myoblast cultures were transiently transfected using liposomes with an expression plasmid encoding full-length, HA-tagged RGS12 prior to being switched into differentiation medium (DMEM, 2% horse serum) when at 80% confluence and cultured for an additional 2 days. Cell cultures were then fixed, permeabilized, and stained with anti-MHC (MF20; *red*). Nuclei were visualized with DAPI (*blue*). *Inset*: Ectopic expression of HA-RGS12 in *Rgs12*^*Δ5-8/Δ5–8*^ myoblasts was confirmed by immunoblotting with anti-RGS12 antibody. **(E)** Differentiation of myoblasts to myotubes *ex vivo*, from micrographs as depicted in panels B and D, was quantitated by the fusion index; statistical significance was tested by one-way ANOVA: ***, p<0.0001, RGS12-null myoblasts exhibited a significantly lower fusion index compared to wild-type, *Rgs12*^+/+^ myoblasts; ###, p<0.0001, *Rgs12*^*Δ5-8/Δ5–8*^ myoblasts expressing HA-RGS12 showed statistically significant recovery of fusion index as compared to mock-transfected *Rgs12*^*Δ5-8/Δ5–8*^ myoblasts. (There was no statistically significant difference between the fusion index of wild-type myoblasts and *Rgs12*^*Δ5-8/Δ5–8*^ myoblasts re-expressing RGS12).

We recently produced a mouse strain constitutively deficient in RGS12 expression and used it to investigate the role of RGS12 in primary myoblast cell fate. This mouse strain is a conventional (*i*.*e*., constitutive) knockout of *Rgs12* exons 5–8 and, as previously reported [32], mice homozygous for the *Rgs12* knockout (*Rgs12*^*Δ5-8/Δ5–8*^) are grossly normal, fertile, and show Mendelian inheritance; an initial survey conducted by UNC’s Behavioral Phenotyping Core (Dr. Sheryl Moy, director) revealed no overt deficits in motor, reflexive, or sensory abilities. Consistent with results from RGS12-directed RNAi in C2C12 cells (Fig 4F–4I), primary myoblasts isolated from *Rgs12*^*Δ5-8/Δ5–8*^ mice (Fig 5A) exhibit significantly reduced myoblast-to-myotube differentiation after 48 hours of *ex vivo* culture in reduced serum-containing medium (Fig 5B). After two days in differentiation medium, *Rgs12*^*Δ5-8/Δ5–8*^ primary myoblast cultures exhibit an approximately 50% reduction in myogenin induction, as compared with wild-type (*Rgs12*^*++/+/+*^) primary myoblast cultures treated in the same fashion (Fig 5C); however, Pax7 and Myf5 reduction, and MRF4 induction, were unchanged. *Rgs12*^*Δ5-8/Δ5–8*^ myoblasts transfected with an RGS12 expression vector and subsequently cultured in reduced serum-containing medium exhibited levels of fusion indistinguishable from those of wild-type myoblasts (Fig 5D).

### Tissue-restricted RGS12 loss leads to normal skeletal muscle development but delays repair

**Fig 6:**
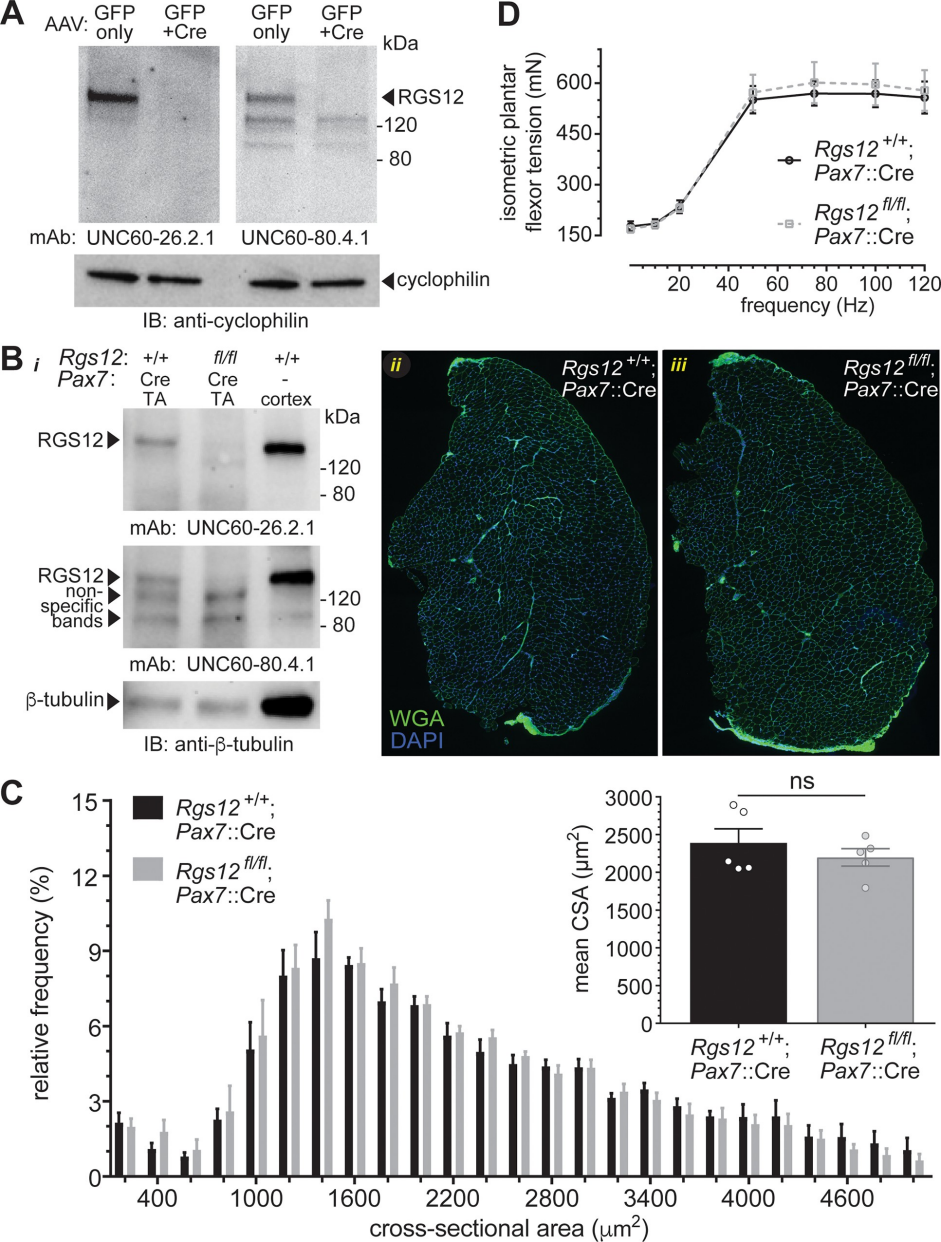
Conditional RGS12 deficiency leads to normal skeletal muscle development. **(A)** Confirmation of Cre-dependent loss of the *Rgs12* gene using a conditional knockout (‘floxed allele’) strategy. *Rgs12*^*fl/fl*^ mouse-derived primary myoblasts were cultured *in vitro* and infected with adeno-associated virus (AAV) expressing green fluorescent protein (GFP only) or Cre recombinase (GFP+Cre). Evidence of Cre recombinase-mediated excision of the *Rgs12* gene (and hence loss of RGS12 expression) was obtained by immunoblotting whole cell lysate from AAV-infected myoblast cultures with indicated anti-RGS12 monoclonal antibodies (each generated by the Siderovski lab and deposited in the Developmental Studies Hybridoma Bank [Iowa City, IA]). **(B)** (i) Western blot confirmation of RGS12 expression in the tibialis anterior (TA) muscle of wild-type mice, Pax7::Cre-dependent loss of RGS12 in the TA of *Rgs12*^fl/fl^ mice, and expression of RGS12 in the cortex (brain) of wild-type mice using two independent monoclonal RGS12 antibodies. (ii, iii) Representative scanning micrographs of TA muscles from wild-type and *Rgs12*^fl/fl^; *Pax7*::Cre mice labeled with wheat-germ agglutinin (WGA; *green*) and nuclear DNA (*blue*). **(C)** Relative frequency distribution of the cross-sectional area (CSA) of muscle fibers as measured by EVOS FL Auto software based on WGA staining (>800 fibers/mouse). No differences in fiber CSA were found between genotypes. *Inset*: The mean CSA of muscle fibers was not different in *Rgs12*^*fl/fl*^; *Pax7*::Cre mice (n = 5) and wild-type *Pax7*::Cre mice (n = 5) as assessed by a Student’s t-test (ns, not significant; p > 0.05). Data are presented as mean ± SEM. **(D)** Tension-frequency display of electrically stimulated isometric plantar flexor contractions at the indicated frequencies. Mean ± SEM tension are displayed of *Rgs12*^fl/fl^; *Pax7*::Cre and wild-type littermate control mice (n = 5/group). A 2-way ANOVA indicated no difference in the tension of wild-type and *Rgs12*-deficient mice at the indicated frequencies.

We recently generated a second mouse strain to complement the constitutive *Rgs12*^*Δ5-8/Δ5–8*^ mouse: specifically, a mouse strain harboring a floxed allele of the *Rgs12* gene that allows for Cre recombinase-mediated excision of the RGS domain-encoding exons 5 and 6 that are flanked by *loxP* sites [32]. This second strain allows for tissue-specific, conditional knockout of RGS12 function. Preparations of primary myoblasts from *Rgs12*^*fl/fl*^ P9 neonates were infected with GFP-tagged Cre recombinase- or GFP-transducing (negative control) adeno-associated viruses (obtained from the U. Penn. Virology Core Facility) and subsequently analyzed for RGS12 expression by immunoblot analysis. Viral transduction of Cre recombinase, but not of GFP alone, resulted in undetectable levels of RGS12 protein expression (Fig 6A).

To help identify an *in vivo* role for RGS12 specifically within skeletal muscle satellite cells, we cross-bred our *Rgs12*^*fl/fl*^ mice with a *Pax7*::Cre driver strain (*Pax7* ^*tm1(cre)Mrc*^ /J). This latter strain [73] retains normal *Pax7* function while expressing Cre recombinase under the control of the *Pax7* promoter in skeletal muscle satellite cells [74–76]. Using two, independent, monoclonal RGS12 antibodies (Fig 6Bi), we confirmed the presence of RGS12 protein in the TA muscle of wild-type mice, but RGS12 was absent in *Rgs12*^fl/fl^; *Pax7*::Cre mice. As an additional control for the specificity of these antibodies, we confirmed RGS12 protein in the cortex of wild-type mouse brain (Fig 6Bi), as previously reported [32].

Mice lacking RGS12 in *Pax7*+ satellite cells were observed to be grossly normal and, when bred from heterozygous crosses (*i*.*e*., *Rgs12*^fl/+^; *Pax7*::Cre *× Rgs12*^fl/+^; *Pax7*::Cre), were born in a normal Mendelian ratio of 1:2:1. *Rgs12* expression in satellite cells did not appear to be necessary for myogenesis during development under normal conditions, as skeletal muscle from *Rgs12*^*fl/fl*^*; Pax7*::Cre mice appear identical to wild-type muscles in their spectrum of fiber cross-sectional area (CSA) (Fig 6Bii, iii and 6C). Additionally, RGS12 deficiency did not impair *in vivo* isometric plantar flexor force generation under normal conditions relative to wild-type mice (Fig 6D). This observation of normal skeletal muscle development in *Rgs12*^*fl/fl*^*; Pax7*::Cre mice is consistent with the lack of overt body-plan and motoric deficits within the conventional *Rgs12*^*Δ5-8/Δ5–8*^ knockout mice as mentioned above.

**Fig 7:**
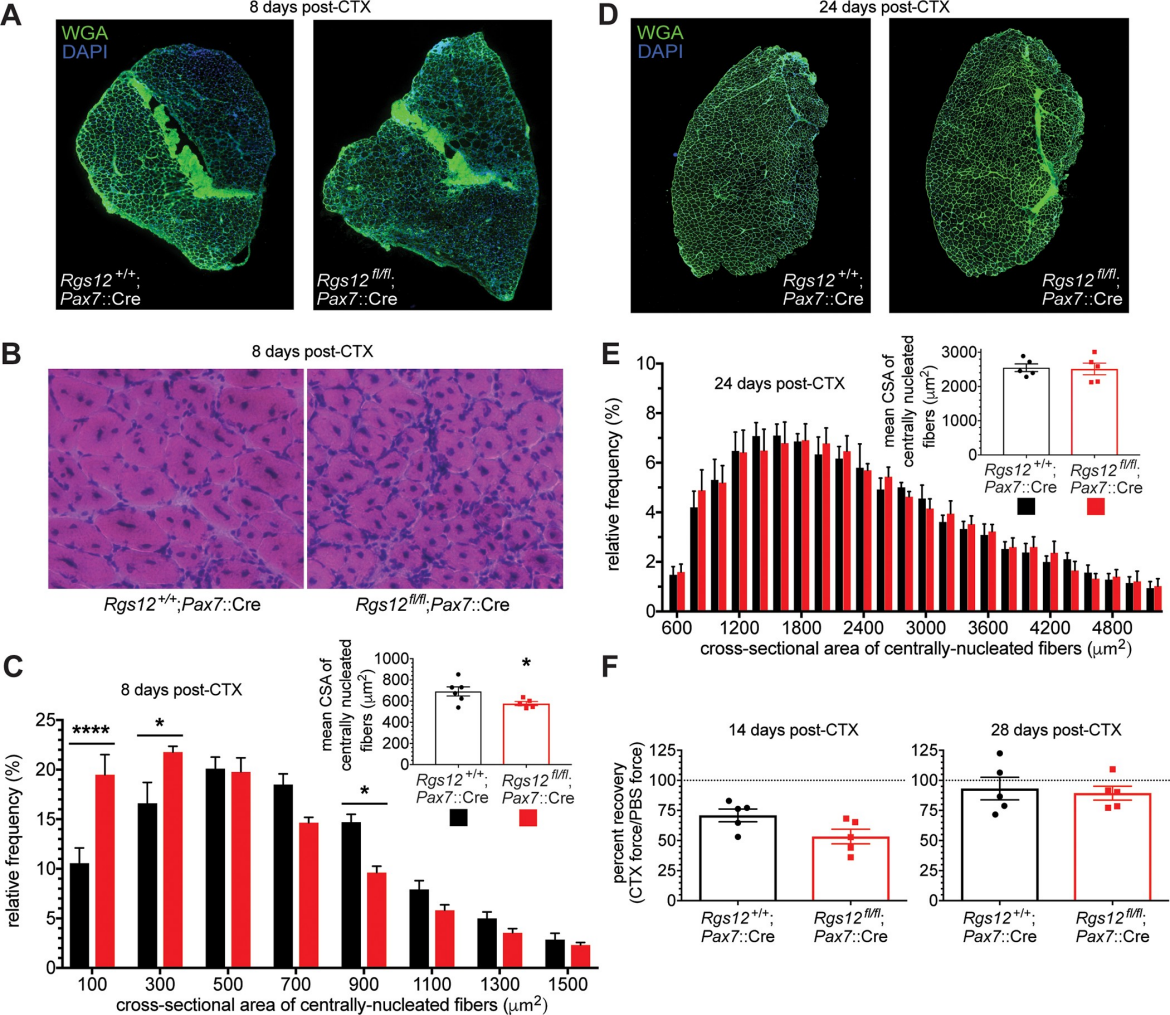
Loss of RGS12 leads to a transient impairment in muscle repair. **A**, Representative cross-sections of tibialis anterior (TA) muscle from indicated mice, labeled 8 days after cardiotoxin (CTX) injection with wheat-germ agglutinin (WGA; *green*) and nuclear DNA (*blue*). **B**, Representative micrographs of H&E-stained TA muscle 8 days after CTX-induced injury. **C,** Relative frequency distribution of the cross-sectional area (CSA) of regenerating (*i*.*e*., centrally-nucleated) muscle fibers 8 days after CTX injection as measured by EVOS FL Auto software based on WGA staining (>800 fibers/mouse). Differences between *Rgs12*^*fl/fl*^; *Pax7*::Cre mice (n = 5) and wild-type *Pax7*::Cre mice (n = 6) were evaluated using a two-way ANOVA with Sidak’s *post hoc* test. ****, p<0.0001; *, p<0.05. *Inset*: The mean CSA of centrally nucleated fibers was shifted towards smaller area in *Rgs12*^*fl/fl*^; *Pax7*::Cre mice *vs* wild-type *Pax7*::Cre mice as assessed by a Student’s t-test; *, p<0.05. Data are presented as mean ± SEM. **D**, Representative cross-sections of TA muscle from indicated mice, labeled 24 days after CTX injection with WGA (*green*) and nuclear DNA (*blue*). **E,** CSA of regenerated myofibers in TA muscle of *Rgs12*^*fl/fl*^; *Pax7*::Cre mice (n = 5) and wild-type *Pax7*::Cre mice (n = 5) 24 days after CTX injury were measured by EVOS FL Auto software based on WGA staining. No differences in fiber CSA were found between genotypes. *Inset*: The mean CSA of centrally nucleated fibers was not different in *Rgs12*^*fl/fl*^; *Pax7*::Cre mice (n = 5) and wild-type *Pax7*::Cre mice (n = 5) as assessed by a Student’s t-test. Data are presented as mean ± SEM. **F.** The maximal tetanic isometric plantar flexor tension of *Rgs12*^*fl/fl*^; *Pax7*::Cre mice (n = 5) and wild-type *Pax7*::Cre mice (n = 5) was obtained in both CTX and PBS treated legs at 14 and 28 days following injection. 14 days post-injection *Rgs12*^*fl/fl*^; *Pax7*::Cre mice trended towards more impairment in the CTX injected leg relative to wild-type mice, as evaluated by a Student’s t-test (p = 0.06). By 28 days post-CTX injection, both genotypes had recovered to an equivalent level relative to their PBS-injected leg. Data are presented as mean ± SEM.

Within *Rgs12*^*fl/fl*^*; Pax7*::Cre muscles, injected with the skeletal muscle-damaging agent CTX and allowed to repair for eight days, there was a marked reduction in the CSA of centrally nucleated regenerating myofibers compared with CTX-injected wild-type muscles (Fig 7A–7C), consistent with the idea that a defect in skeletal muscle repair is engendered by RGS12 loss. Additionally, functional muscle recovery was perturbed in RGS12-deficient mice 14 days following CTX injection. *In vivo* plantar flexor force generation was impaired following CTX-induced injury (relative to the contralateral uninjured leg) in both wild-type and *Rgs12*^*fl/fl*^*; Pax7*::Cre mice, but function trended towards being more impaired in RGS12-deficient mice (Fig 7F). However, it appears that skeletal muscle repair is only delayed in *Rgs12*^*fl/fl*^*; Pax7*::Cre mice. By 24 days following CTX injection, the CSA of centrally nucleated myofibers were equivalent to those of wild-type mice (Fig 7D and 7E). Additionally, 28 days after injury plantar flexor function was equivalently recovered in wild-type and *Rgs12*-deficient mice (Fig 7F).

### Tissue-restricted loss of RGS12 does not alter the dystrophic phenotype of *mdx* mice

**Fig 8:**
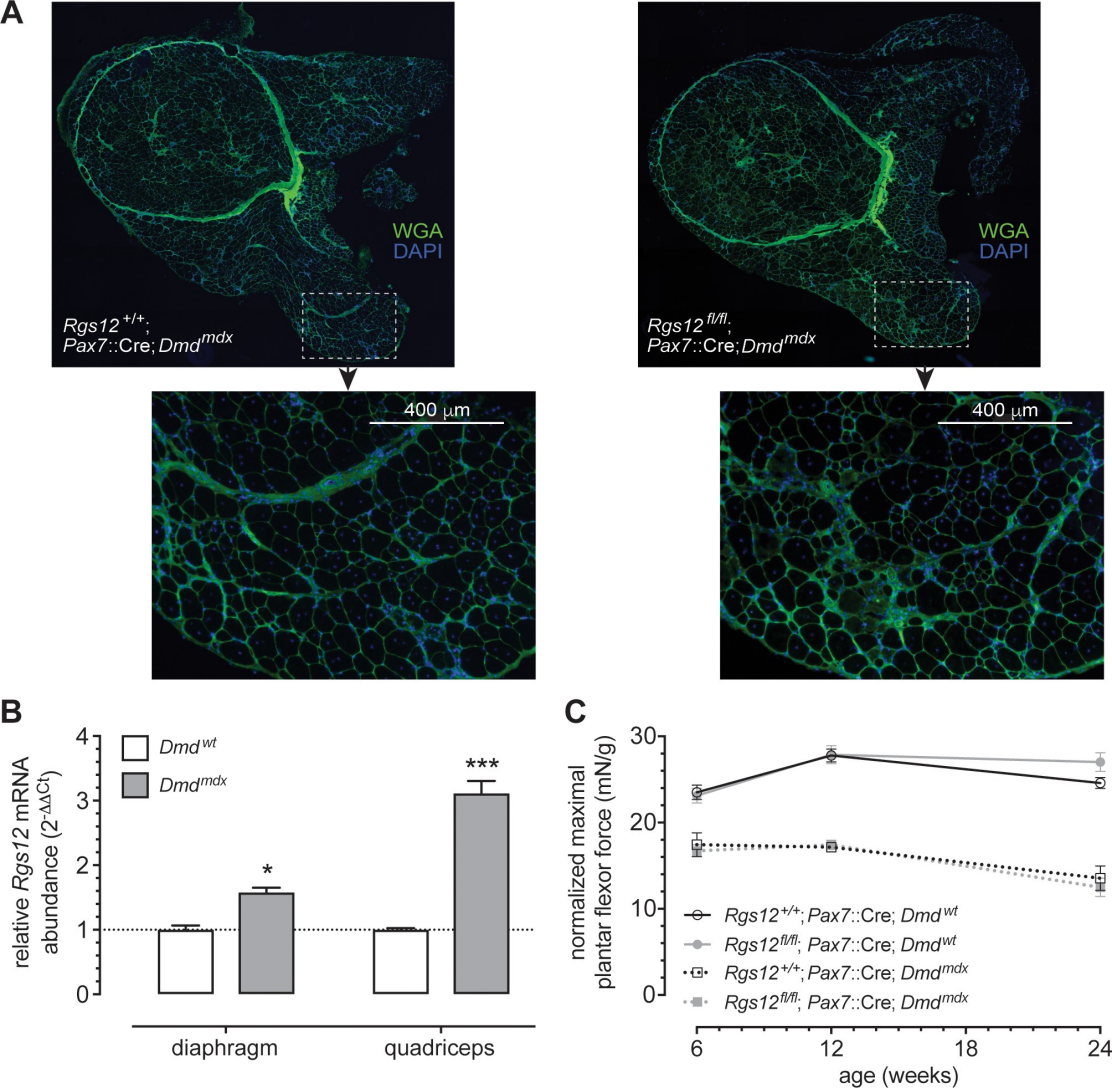
*Rgs12* mRNA is upregulated in skeletal muscles of *Dmd*^*mdx*^ mice, but RGS12 loss does not appear to alter the muscle wasting phenotype of the *Dmd*^*mdx*^ mouse model. **A**, Representative micrographs of quadriceps femoris muscle from indicated mice, labeled with wheat-germ agglutinin (WGA; *green*) and nuclear DNA (*blue*). Loss of RGS12 in satellite cells does not alter skeletal muscle morphology of *mdx* mice. **B**, *Rgs12* expression was elevated in the diaphragm (*, p<0.05) and quadriceps femoris (***, p<0.001) muscles of *mdx* mice as assessed by qRT-PCR analysis. Bar graph displays the mean ± SEM; differences between wild-type and *mdx* mice were shown to be statistically significant by Student’s t-test. **C**, The maximal tetanic isometric plantar flexor tension normalized to body weight of *Rgs12*^*fl/fl*^; *Pax7*::Cre mice (n = 5), wild-type *Pax7*::Cre mice (n = 5), *Rgs12*^*fl/fl*^; *Pax7*::Cre; *mdx* mice (n = 5), and wild-type *Pax7*::Cre; *mdx* mice (n = 5) obtained at 6, 12, and 24 weeks of age are displayed as mean ± SEM. A two-way ANOVA indicated *mdx* mice had impaired muscle function relative to *Dmd* wild-type mice at each age; however, *Rgs12* deficiency did not alter muscle function in *mdx* or *Dmd* wild-type mice.

In addition to assessing the expression of *Rgs12* in response to pharmacological muscle damage, we also examined expression of *Rgs12* in dystrophic muscles of *mdx* mice (Fig 8). *Rgs12* expression was found to be elevated in the quadriceps and diaphragm muscles of 12-week-old *mdx* mice relative to wild-type controls (Fig 8B). To determine whether RGS12 influences the progression of the dystrophic phenotype, we generated *mdx* mice conditionally lacking RGS12 in *Pax7*+ satellite cells (*i*.*e*., *Rgs12*^fl/fl^; *Pax7*::Cre; *Dmd*^mdx^ mice). We examined the morphology of quadriceps muscles from both *Rgs12*^fl/fl^; *Pax7*::Cre; *Dmd*^mdx^ and *Rgs12*^+/+^; *Pax7*::Cre; *Dmd*^mdx^ mice at 24 weeks of age. Lack of RGS12 in satellite cells did not overtly alter the dystrophic phenotype in *mdx* mice (*e*.*g*., Fig 8A). To determine whether RGS12 deficiency alters the functional deficits normally witnessed within the *mdx* mouse model, we examined isometric plantar flexor force generation at six, 12, and 24 weeks of age. A clear deficit was found in the normalized maximum force generated by *mdx* mice at each age assessed; however, RGS12 deficiency did not alter this deficit (Fig 8C). These data suggest that loss of RGS12 may be compensated for within model syndromes of prolonged bouts of muscle damage and repair. Future experiments adding frank CTX-induced injury to muscle within *Rgs12*^fl/fl^; *Pax7*::Cre; *Dmd*^mdx^ mice would be worthwhile to perform to test the extent of such compensation.

## Conclusions

The results presented herein, from both cell culture and mouse model studies, illuminate a previously underappreciated role of RGS12 in muscle precursor-cell function. Our original report [10], that RGS12 expression is temporospatially regulated in the developing mouse embryo, with strong expression in developing skeletal muscle, was highly suggestive evidence that fueled these present studies. We have since found *Rgs12* expressed in mouse adult TA muscle, and its expression increased following injury and in dystrophic muscle, further suggesting a role for RGS12 in muscle regeneration. The dynamic regulation of *Rgs12* mRNA expression seen in muscle precursor cells during myogenesis is consistent with a potential role for the encoded G protein- and MAPK-scaffold in coordinating myogenic signals: *Rgs12* is expressed in satellite cells and proliferating myoblasts, and reduced as myoblasts undergo differentiation and fusion into myotubes. Moreover, RGS12 protein is seen to bind to endosomally enriched phosphoinositides *in vitro* and to be localized to early endosomes in cultured myoblasts, suggesting that its scaffolding properties [7] may localize specific molecular interactions (*i*.*e*., Ras/Raf/MEK/ERK signaling cascade components) to certain cytosolic sub-regions within the activated satellite cell itself, the trans-amplifying cell destined for differentiation, or both.

We demonstrate here that ectopically expressed, oncogenic (G12V) H-Ras associates with (and stabilizes the levels of) RGS12 in transfected C2C12 cells. These observations are consistent with prior demonstrations of an association of RGS12 with activated but not wild-type H-Ras [7]; yet, on their own, they do not establish causality for the known or suspected functions of either protein. The inhibition of myotube formation seen upon activated H-Ras expression [24–26] (Fig 3) likely arises from a pleotropy of signaling consequences downstream of multiple H-Ras effectors [77] in addition to, or even exclusive of, RGS12 function as an H-Ras/Raf/MEK2 scaffold [7]. Moreover, the stabilization of endogenous RGS12 levels upon activated H-Ras over-expression may be an effect, rather than the cause, of an inhibited differentiation program. The inhibition of C2C12 myotube formation seen upon *Rgs12* knockdown and enhanced formation upon ectopic RGS12 over-expression, while seemingly contradictory to the stabilized RGS12 levels seen in the poorly differentiating C2HRas cell line, are both likely to be artificial outcomes of the “combinatorial inhibition” phenomenon commonly observed with signaling scaffolds [78, 79], as we have previously denoted in explaining biphasic effects of RGS12 expression in studies of receptor-initiated ERK activation [7].

Our present *in vitro* analyses demonstrate that RGS12 over-expression in myoblasts represses proliferation and promotes differentiation, suggesting that RGS12 may normally act to coordinate GTPase and/or MAPK signals downstream of pro-differentiation and/or pro-fusion external input(s) (perhaps *via* Wnt-liganded and/or BAI3 GPCR activation(s); refs. [80, 81]) and thereby impact on the terminal differentiation of myoblasts, similar to how RGS12 (and the related RGS14) is thought to act in neuronal axonogenesis driven by NGF and bFGF signaling [7, 37]. Consistent with the *in vitro* findings, delayed muscle regeneration was observed in mice conditionally deficient in *Rgs12* expression in *Pax7*+ satellite cells; however, muscle repair returned to normal by 24 days post-injury. When tissue-restricted *Rgs12* loss was cross-bred into the *mdx* mouse model of Duchenne muscular dystrophy, no change in the dystrophic phenotype was observed over time. Collectively, these data support the hypothesis that RGS12 plays a role in coordinating signals during myogenic programming, but its loss can be compensated for over time and in models of repeated muscle damage and repair. Given that RGS proteins are generally considered “regulators” of the potency and amplitude of receptor-initiated intracellular signaling (*e*.*g*., refs. [82, 83]), rather than obligate components thereof, the most likely mechanism of such compensation is achievement of a particular threshold of accumulated signal over time in the absence of RGS12’s regulatory influence. Further elucidating both time- and tissue-restricted loss of RGS12 in adult skeletal muscle satellite cells should provide improved resolution as to the *in vivo* role(s) of RGS12 in myogenic signaling. For example, a further test for a specific role of RGS12 in muscle wasting could entail studies of muscle progenitor phenotype and function with *Utrn*^*tm1Ked*^*; Dmd*^*mdx*^*; Rgs12*-deficient mice. Utrophin (Utrn), a cytoskeletal protein highly related to dystrophin, is thought to compensate for dystrophin loss in the spontaneous *mdx* mouse model of DMD. While utrophin-deficient mice are healthy and generally show no signs of weakness [84], utrophin/dystrophin-deficient mice exhibit a more severe dystrophic phenotype than single *Dmd*^*mdx*^ mutant mice [85]: *i*.*e*., the double knockout strain exhibits a phenotype that more closely resembles DMD in humans with severe progressive muscular dystrophy resulting in premature death. Recent evidence [86] suggests that this accelerated disease progression is associated with a more rapid decrease in the number and regenerative potential of muscle progenitor cells; these results highlight the notion that alleviating progenitor cell depletion could delay the onset of histopathology seen in DMD patients [86]. Our hope is that future findings as to the role(s) of RGS12 in muscle repair and muscle wasting could inform novel therapeutic strategies for DMD and other muscular dystrophies.
